# Associations between improvements in psychological variables and subsequent sick leave among persons receiving a multimodal intervention for exhaustion disorder

**DOI:** 10.1186/s12889-023-16799-x

**Published:** 2023-10-11

**Authors:** Jakob Clason van de Leur, Monica Buhrman, Kajsa Wallby, Amanda Karlström, Fred Johansson

**Affiliations:** 1 Department of Psychology, Uppsala University, Box 1225, Uppsala, 751 42 Sweden; 2 Department of Health Promotion Science, Sophiahemmet University, Valhallavägen 91, SE- 114 28 Stockholm, Sweden

**Keywords:** Exhaustion disorder, Clinical burnout, Work stress, Return to work, Sick leave

## Abstract

**Background:**

The incidence of sick leave due to stress-related disorders such as exhaustion disorder (ED) is high in many economically developed countries. Meanwhile, knowledge about facilitating return to work during clinical interventions for ED patients is still limited. The current study aimed to investigate if improvements in exhaustion symptoms, insomnia, perfectionistic behaviors, psychological flexibility, and perceived work ability during treatment of ED were associated with subsequent sick leave in the year following treatment.

**Methods:**

Using a cohort of 880 ED patients who had participated in a multimodal intervention based on Cognitive Behavior Therapy, we estimated the association between one standard deviation (SD) improvement in treatment-related variables and the rate of net days of sick leave one-year following treatment.

**Results:**

Our results showed that improvements in all treatment-related variables were associated with lower sick leave rates one year following treatment. Improvements in exhaustion symptoms (rate ratio (RR): 0.70 [95% CI 0.66; 0.75]) and self-perceived work ability (RR 0.56 [95% CI 0.50; 0.63]) showed the strongest associations to subsequent sick leave.

**Conclusions:**

These findings suggest that interventions focusing on exhaustion symptoms, insomnia, perfectionistic behaviors, psychological flexibility, and perceived work ability can have a meaningful impact on ED patients’ subsequent sick leave.

**Trial registration:**

Clinicaltrials.gov (Identifier: NCT03360136).

**Supplementary Information:**

The online version contains supplementary material available at 10.1186/s12889-023-16799-x.

## Introduction

The prevalence of stress-related disorders is rising in many economically developed countries [1, 2], posing a significant economic burden due to loss of productivity [3]. Besides having a considerable socioeconomic impact, long-term sick leave due to mental health diagnoses is associated with a higher risk of suicide and overall mortality [4]. Additionally, persons on sick leave report experiencing several negative consequences related to being on sick leave, such as deteriorated mental health, fewer social contacts, and less income [5, 6]. Consequently, there is an apparent need for effective clinical interventions that facilitate return to work for those suffering from stress-related disorders.

For many people, exposure to persistent non-traumatic stress leads to a debilitating state of exhaustion not equivalent to depression [7]. In Sweden, the diagnosis of stress-induced exhaustion disorder (ED; International Classification of Diseases 10 code F43.8 A) is utilized to label this condition. ED is signified by substantial debilitating symptoms of exhaustion coupled with cognitive impairments and can be described as the end stage of a severe burnout process requiring professional healthcare interventions [7]. Although there is no consensus regarding the terminology for exhaustion due to persistent non-traumatic stress, several international publications suggest that ED is not an uniquely Swedish condition [2, 8, 9]. In Sweden, ED now accounts for a large part of all long-term sick leave [10]. Consequently, the need for research on supporting work resumption for ED patients has been highlighted, as the recovery process is often protracted [11]. Recently, a systematic review of return-to-work (RTW) interventions for workers with common mental disorders showed that multi-component interventions, including workplace contact and graded RTW, leads to a decrease in sick leave rates [12]. However, knowledge of methods to reduce sick leave due to ED specifically is still limited [13].

Three reviews on RTW interventions for ED conclude that the current body of literature is too limited for any definitive conclusions but those interventions focusing on a workplace dialogue with the employer, and Cognitive Behavior Therapy (CBT) incorporating methods focusing on fostering work resumption and coping with work stressors (work-focused CBT), show promising results [13–15]. Three recent publications have focused on interventions to reduce sick leave due to ED. First, Finnes et al. (2019) compared workplace dialogue to Acceptance and commitment therapy (ACT) and a combination of both in a mixed sample with mostly ED patients (66% of ttal sample, *n* = 234). No differences in sick leave rates were identified at follow-up [16]. Secondly, in a similar design with a sample of patients with ED and adjustment disorder, Salomonsson et al. (2020) found no significant differences in sick leave days when comparing work-focused CBT with regular CBT or a combination of both [17]. Finally, in a three-armed randomized controlled trial of patients with stress-related disorders, Hoff et al. (2022) compared an integrated intervention combining extensive vocational rehabilitation with mental healthcare (arm one) to mental healthcare with a standardized vocational intervention (arm two) and service as usual (arm three). Surprisingly, arms one and two with vocational interventions resulted in increased sick leave rates compared to service as usual. Consequently, work is needed to help determine what methods should be utilized in ED treatment to target work resumption and reduce sick leave more effectively [18].

A plausible hypothesis concerning the underlying processes of work resumption is that reductions in symptom burden during psychological treatment mediate RTW-outcomes and that a decrease in symptoms associated with the targeted condition eventually leads to a reduction in sick leave [14]. To our knowledge, the relationship between symptom reduction and sick leave has not been empirically examined for persons undergoing treatment for ED. Another important goal in aiding people in an RTW process is increasing their perceived work ability. Generally, a low degree of perceived work ability is associated with increased risk of long-term sick leave [19]. Perceived work ability and degrees of self-rated burnout have been shown to be predictors of work resumption in patients on long-term sick leave [20]. Furthermore, previous research on ED indicates that decreases in insomnia are associated with improved exhaustion symptoms and increased chances of RTW [13]. Therefore, decreasing exhaustion symptoms and insomnia and increasing perceived work ability potentially seem to be putative targets of ED treatment to reduce sick leave rates.

Other treatment targets potentially linked to sick leave could be perfectionism and psychological flexibility. Perfectionism is characterized by the striving for excessively high standards and concerns about not meeting those standards, conceptualized by two main dimensions perfectionistic strivings and perfectionistic concerns [21]. Patients with ED often seem to struggle with high perfectionism and over-commitment [22]. Furthermore, published treatment trials of ED treatment often describe components specifically targeting perfectionism [23].

Other treatment components often described in clinical trials of ED are mindfulness exercises and the identification of core life values - components associated with ACT [16]. ACT focuses on increasing psychological flexibility, a construct described as “the capacity to persist or change behavior in a way that includes conscious and open contact with internal experiences, together with an appreciation of what each situation entails to one’s values and goals” [24]. Low degrees of psychological flexibility have been shown to predict work absenteeism over time [25].

As part of a larger single-armed open clinical trial, we have previously shown symptom reduction and reduced sick leave rates in a large sample of ED patients (N = 389) participating in a standardized CBT-based multimodal intervention (MMI). In addition to specific vocational measures, the MMI included components targeting the putative target variables described above, potentially important in reducing sick leave rates over time. Therefore, the current study aimed to investigate if improvements in exhaustion symptoms, insomnia, perfectionistic behaviors, psychological flexibility, and perceived work ability during treatment of ED were associated with fewer net days of sick leave in the one-year period following treatment. More specifically, our hypotheses were as follows: (1) Reduced exhaustion symptoms, insomnia, and perfectionism during treatment are associated with lower sick leave rates in the year following MMI. (2) Increased perceived work ability and psychological flexibility are associated with lower sick leave rates in the year following MMI.

## Methods

### Participants and design

We used data from a clinical cohort following patients receiving a 24-week Multi-modal intervention (MMI) for exhaustion disorder at two healthcare clinics in Stockholm, Sweden. Both clinics were part of a specialized healthcare program called “The health care.

choice for treatment of longstanding pain with or without comorbidity, and ED”, administered on behalf of the Health Care Services Stockholm County. Measurements were taken at five time-points: assessment, pre-treatment (week 0), mid-treatment (week 12), post-treatment (week 24) and a follow-up 12 months after completing treatment. To decrease instrumentation bias, the order of the questionnaires was randomized at each dispatch.

All participants were referred for exhaustion disorder from primary health care centers, general practitioners, and occupational health services. Before entering the cohort, all potential participants were assessed by multi-professional teams (physician, psychologist and physiotherapist). To be included in the cohort, the patients needed to (1) be diagnosed with exhaustion disorder by the multi-professional team, (2) score > 4.5 on the Shirom-Melamed Burnout Questionnaire [26], a cut-off predetermined by the Health Care Services Stockholm County for being accepted to the health care initiative mentioned above (see previous section); (3) be considered suitable for group treatment, (4) be 18–65 years of age, (5) not abuse alcohol or drugs, (6) not participate in any other MMI, (7) not have severe depression, moderate/high risk of suicide or untreated PTSD. Data collection was registered on Clinicaltrials.gov (Identifier: NCT03360136). Furthermore, this study was approved by the Regional Ethical Review Board in Stockholm, Sweden (Approval Nr. 2016/1834-31/2) and followed the ethical principles of the Declaration of Helsinki. All participants provided informed consent before entering the study. In short, the 24-week MMI was centered on CBT involving approximately 1–3 weekly sessions. These sessions encompassed group and individual elements and incorporated vocational measures such as rehabilitation meetings with the employer. For more information on the inclusion process and the contents of the MMI, see [27].

In total, 1643 persons were assessed between September 2017 and Mars 2019. Out of these, 472 did not fulfill the criteria for the MMI, 15 were included in a rehabilitation program for pain, and 73 were offered a short version of the rehabilitation program for ED (12 or 16 weeks). Of the 1083 included in the MMI, 151 declined to participate in the study, 17 withdrew before the start of MII, and 27 withdrew during MII. Further, 8 participants were excluded since they were on disability pension and could not be granted sick leave (based on registry data collected one year after treatment completion), leaving 880 participants in the cohort.

### Measures

#### Outcome

Net days of sick leave in the year following treatment were obtained from registers from the Swedish Social Insurance Agency (SSIA), which is the governmental agency that administers reimbursement for sick leave longer than 14 days. SSIA grants both full-time and part-time sick leave (i.e. 0%, 25%, 50%, 75% or 100%). We obtained information on paid sick leave from SSIA six months prior to treatment to one year following treatment for all participants. To calculate net days of sick leave in the year following treatment, we summed all days of sick pay in the year following the first calendar months after treatment completion. Part-time sick days were treated as fractions when summing (e.g. one day on 25% paid sick leave was counted as 0.25 days).

#### Exposures

Exhaustion symptoms were measured using the Karolinska Exhaustion Disorder Scale (KEDS; [28]). KEDS consists of nine items, covering the Swedish diagnostic criteria for exhaustion disorder, each rated on a 7-point scale from 0 to 6. The total score is calculated by summing all items giving a score ranging from 0 to 54. KEDS has shown good validity (discriminating against healthy controls) and satisfactory internal consistency in earlier evaluations [28]. Cronbach’s alpha for KEDS in the current sample was 0.75.

Insomnia symptoms were measured using the Insomnia Severity Index (ISI), consisting of seven items rated on a five-point numerical scale from 0 to 4, and the items are summed to give a total score ranging from 0 to 28. The ISI has demonstrated adequate internal consistency and sensitivity to changes in insomnia severity [29]. Cronbach’s alpha in the current sample was 0.85.

Psychological flexibility was measured using the Swedish Acceptance and Action Questionnaire (SAAQ-II), consisting of six items rated on a seven-point numerical scale from 1 (Never true) to 7 (Always true). The items are summed to give a total score ranging from 6 to 42, where a low total score signifies a high degree of psychological flexibility. The SAAQ-II has shown adequate convergent validity and internal consistency in psychometric evaluations [30], and Cronbach’s alpha in the current sample was 0.86.

Perfectionistic concerns and perfectionistic strivings were measured using the Clinical Perfectionism Questionnaire (CPQ). The CPQ consists of 12 items rated on a numerical scale from 1 (Never) to 4 (Always). Factor analytic studies have indicated two dimensions of the CPQ, perfectionistic concerns (item 1, 3, 6, 10 and 11) and perfectionistic strivings (items 4,5,7,9 and 12) [31]. We summed the items for each dimension respectively, giving subscale scores ranging from 4 to 20. Cronbach’s alpha was 0.79 for the perfectionistic concerns’ subscale and 0.66 for the perfectionistic strivings’ subscale in the current sample.

Self-perceived work ability was measured with the following item: “Let us assume that your work ability when it was at its peak, was rated 10. What number would you give your current work ability?“. This item is rated on an 11-point scale ranging from 0 (cannot work at all) to 10 (my work ability at its peak). This question was taken from the Work Ability Index (WAI) and has been shown to correlate well with the entire scale and is considered a reliable measure to follow the development of a person’s work ability [32].

#### Covariates

Covariates were chosen based on prior knowledge of predictors of sick leave in common mental disorders [33, 34] and we included any factor assumed to potentially cause both exposure and the outcome to control for confounding. All covariates were measured pre-treatment to ensure no covariate was on the causal pathway from the exposures to the outcome.

The covariates included: gender (male, female); age (18–29, 30–41, 42–53, 54–65); civil status (Single, Married/living with partner, Partner not living together); yearly household income in SEK (0-250k, 250k-500k, 500k-1000k, > 1000k); education level (elementary or secondary school, university < 3 years, university > 3 years, other); country of birth (Sweden, Nordic countries, Europe, Outside Europe); type of work (handling of heavy loads, heavy repetitive work, medium-heavy work, light repetitive work, administrative/ computer work); comorbidity (none, psychiatric condition, pain condition, psychiatric and pain conditions); unemployment (yes/no); net days of sick leave six months before treatment (continuous); anxiety and depression symptoms (measured with the Hospital Anxiety and Depression scale treated as two continuous subscales) [35].

### Statistical analyses

**Fig. 1:**
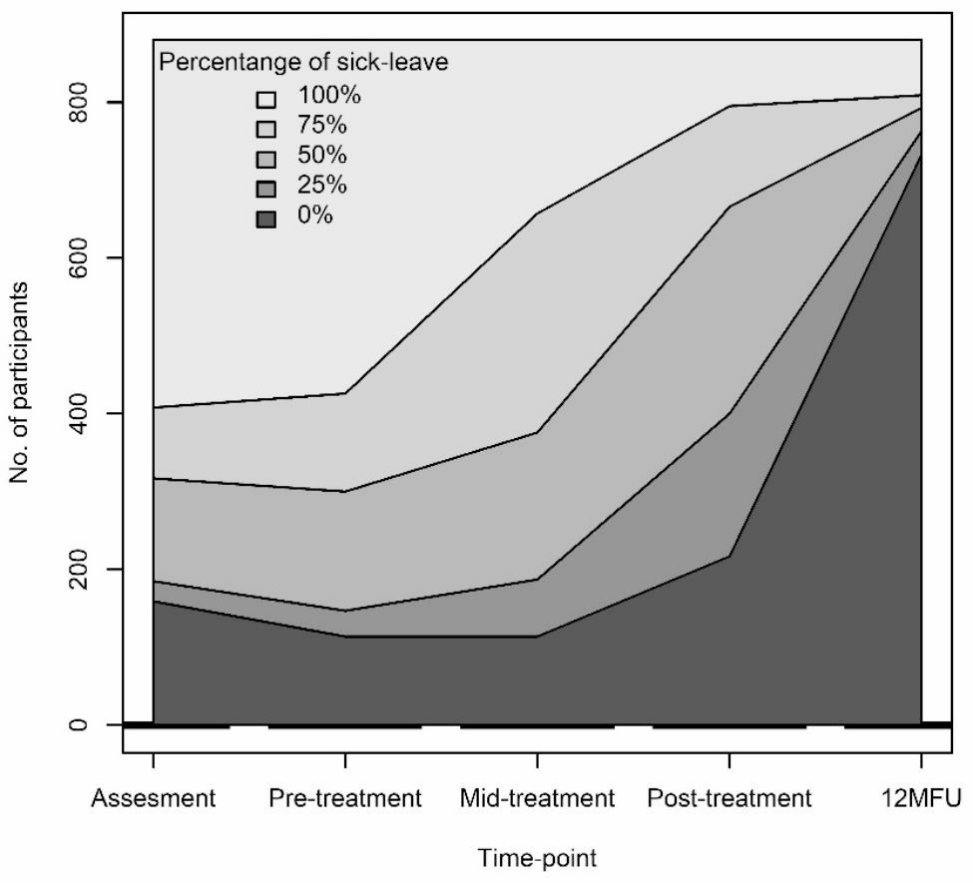
Number of participants on by their point-wise percentage of sick leave across the follow-up period

Baseline characteristics of the sample are presented for the full sample in Table 1, and by quartiles of the exposure variables in Supplemental Tables 1–6. Means and SDs of the exposures at pre- and post-treatment is presented in Table 2. The point-wise amount of net sick leave grated by the SSIA to the participants at each follow-up time-point is presented in Fig. 1.

**Table 1:** Pre-treatment characteristics for the full sample, and by quartiles of KEDS score at post-treatment

		KEDS quartiles at post-treatment			
	Full sample (n = 880)	1st (n = 230)	2nd (n = 210)	3rd (n = 211)	4th (n = 202)
Gender, n (%)					
Male	122 (13.9)	41 (17.8)	23 (11.0)	27 (12.8)	29 (14.4)
Age, n (%)					
18–29 years	70 (8.0)	21 (9.1)	21 (10.0)	14 (6.6)	11 (5.4)
30–41 years	326 (37.0)	85 (37.0)	76 (36.2)	78 (37.0)	77 (38.1)
42–53 years	354 (40.2)	87 (37.8)	80 (38.1)	93 (44.1)	83 (41.1)
54–65 years	130 (14.8)	37 (16.1)	33 (15.7)	26 (12.3)	31 (15.3)
Civil status, n (%)					
Married/living together	557 (63.3)	144 (62.6)	142 (67.6)	133 (63.0)	118 (58.4)
Partner living apart	58 (6.6)	14 (6.1)	12 (5.7)	10 (4.7)	20 (9.9)
Single/Other	265 (30.1)	72 (31.3)	56 (26.7)	68 (32.2)	64 (31.7)
Yearly household income, n (%)					
0–250k SEK	73 (8.3)	16 (7.0)	12 (5.7)	18 (8.5)	26 (12.9)
500k − 1000k SEK	668 (75.9)	176 (76.5)	156 (74.3)	160 (75.8)	155 (76.7)
>1000k SEK	139 (15.8)	38 (16.5)	42 (20.0)	33 (15.6)	21 (10.4)
Education level, n (%)					
Elementary or secondary school	219 (24.9)	55 (23.9)	49 (23.3)	53 (25.1)	56 (27.7)
University less than 3 years	140 (15.9)	39 (17.0)	36 (17.1)	26 (12.3)	34 (16.8)
University 3 years or more	482 (54.8)	124 (53.9)	118 (56.2)	121 (57.3)	105 (52.0)
Other	39 (4.4)	12 (5.2)	7 (3.3)	11 (5.2)	7 (3.5)
Country of birth, n (%)					
Sweden	762 (86.6)	204 (88.7)	185 (88.1)	179 (84.8)	171 (84.7)
The north of Europe	23 (2.6)	7 (3.0)	3 (1.4)	6 (2.8)	7 (3.5)
Europe	34 (3.9)	4 (1.7)	12 (5.7)	10 (4.7)	7 (3.5)
Other country	61 (6.9)	15 (6.5)	10 (4.8)	16 (7.6)	17 (8.4)
Type of work					
Handling of heavy loads	19 (2.3)	6 (2.9)	3 (1.5)	3 (1.5)	7 (3.8)
Heavy repetitive work	40 (4.9)	6 (2.9)	10 (5.0)	11 (5.6)	13 (7.1)
Medium-heavy work	144 (17.7)	32 (15.4)	35 (17.5)	33 (16.7)	41 (22.3)
Light repetitive work	49 (6.0)	13 (6.2)	11 (5.5)	17 (8.6)	7 (3.8)
Administrative/ computer work	562 (69.0)	151 (72.6)	141 (70.5)	134 (67.7)	116 (63.0)
Comorbidity					
No comorbidity	528 (60.0)	144 (62.6)	122 (58.1)	140 (66.4)	104 (51.5)
Comorbid psychiatric disorder	304 (34.5)	74 (32.2)	75 (35.7)	60 (28.4)	87 (43.1)
Comorbid pain disorder	28 (3.2)	8 (3.5)	8 (3.8)	6 (2.8)	5 (2.5)
Comorbid pain and psychiatric disorder	20 (2.3)	4 (1.7)	5 (2.4)	5 (2.4)	6 (3.0)
Employed	786 (93.2)	208 (92.9)	191 (95.5)	185 (93.0)	178 (91.3)
Net days of sick leave 6 months prior to treatment, mean (SD)	91.53 (56.79)	81.93 (50.56)	88.10 (55.96)	96.04 (57.46)	98.33 (60.80)
Depression symptoms, mean (SD)	11.23 (3.68)	10.20 (3.52)	11.05 (3.48)	11.65 (3.75)	12.23 (3.67)
Anxiety symptoms, mean (SD)	11.59 (4.01)	10.86 (3.89)	11.22 (3.77)	11.96 (3.82)	12.36 (4.36)

**Table 2:** Pre- and post-treatment values of the exposures

Predictor	Pre-treatment		Post-treatment,	
	No.	Mean (SD)	No.	Mean (SD)
Exhaustion symptoms, mean (SD)	879	34.8 (6.2)	853	22.7 (8.1)
Insomnia symptoms, mean (SD)	880	16.0 (6.1)	853	9.8 (5.8)
Psychological flexibility, mean (SD)	872	22.5 (6.9)	852	17.8 (6.8)
Perfectionistic concerns, mean (SD)	876	14.5 (3.5)	855	10.9 (3.5)
Perfectionistic strivings, mean (SD)	876	13.2 (3.2)	855	10.5 (3.0)
Self-perceived work ability, mean (SD)	877	2.6 (2.0)	590	5.3 (2.1)

The association between changes in the exposure variables during treatment and the mean number of sick days in the year following treatment was estimated using Generalized linear models (GLM’s) with a log link function and a Gaussian error distribution. Separate models were built for each exposure. To avoid zero-values in the outcome (to enable the use of the log-link), a constant of 0.0001 was added to the net sick day values of 0.

To model the association between changes in the exposure variables during treatment and the subsequent number of net sick days, we estimated the association between the post-treatment levels of the exposure and the outcome conditional on pre-treatment levels of the exposure. By conditioning on pre-treatment values of the exposures, pre-treatment values are held constant, so that the estimates of the post-treatment values of the exposures can be interpreted as the association between of change in the exposures during treatment and net days of sick leave in the year following treatment. Controlling for earlier exposure levels also helps reduce the risk of reverse causation and unmeasured confounding (see [36] for more details on this approach). Separate models were fitted for each exposure taking the form:

$${log}\left\{E\left(Y| {X}_{post}, {X}_{pre}, {C}_{pre}\right)\right\}= {\beta }_{0}+ {\beta }_{X post }{X}_{post}+ {\beta }_{X pre }{X}_{pre}+ {\beta }_{C}^{{\prime }}{C}_{pre}$$

Where *Y* denotes the net days of sick leave in the year following treatment. *X*_*post*_ denotes the exposure variable at post-treatment, *X*_*pre*_ is the exposure variable at pre-treatment, and *C*_*pre*_ is a set of pre-treatment covariates. *β*_*Xpost*_ represents the association between change in the exposure from pre-treatment to post-treatment and net days of sick leave.

We fitted three models for each exposure, using different sets of pre-treatment covariates. Model 1 included only the exposure of interest measured at pre- and post-treatment. In Model 2 we further controlled for gender, age, civil status, yearly household income in SEK, education level, country of birth, type of work, comorbidity, and unemployment. In Model 3, we further controlled for number of days on sick leave six months prior to treatment, anxiety and depression symptoms at pre-treatment and pre-treatment levels of all exposures.

Table 3 presents the rate ratio (RR) of estimated mean sick days corresponding to a one standard deviation *decrease* in the exposure variable during treatment, calculated as ${\varvec{e}}^{- {\varvec{\beta }}_{\varvec{X}\varvec{p}\varvec{o}\varvec{s}\varvec{t}} \varvec{S}\varvec{D}\left(\varvec{X}\varvec{p}\varvec{r}\varvec{e}\right)}$. Estimates are presented for each exposure and for Model 1–3 along with 95% confidence intervals (CI’s).

**Table 3:** Associations between changes in the exposures during treatment and mean net days of sick leave in the year following treatment

	n	RR ^a^ (95% CI)	E-value PE	E-value UCL
Exhaustion symptoms				
Model 1^b^ Model 2^c^ Model 3^d^	852 754 742	0.65 (0.61; 0.69) 0.70 (0.65; 0.75) 0.70 (0.66; 0.75)	2.45 2.21 2.21	2.26 2.00 2.00
Insomnia symptoms				
Model 1^b^ Model 2^c^ Model 3^d^	853 751 740	0.68 (0.60; 0.77) 0.81 (0.72; 0.91) 0.75 (0.67; 0.84)	2.30 1.77 2.00	1.92 1.43 1.67
Psychological flexibility				
Model 1^b^ Model 2^c^ Model 3^d^	845 746 739	0.69 (0.59; 0.82) 0.74 (0.65; 0.83) 0.73 (0.65; 0.83)	2.26 2.04 2.08	1.74 1.70 1.70
Perfectionistic concerns				
Model 1^b^ Model 2^c^ Model 3^d^	851 752 743	0.79 (0.70; 0.88) 0.76 (0.68; 0.84) 0.78 (0.71; 0.86)	1.85 1.96 1.88	1.53 1.67 1.60
Perfectionistic strivings				
Model 1^b^ Model 2^c^ Model 3^d^	851 752 743	0.76 (0.67; 0.87) 0.77 (0.69; 0.86) 0.77 (0.69; 0.86)	1.96 1.92 1.92	1.56 1.60 1.60
Self-perceived work ability				
Model 1^b^ Model 2^c^ Model 3^d^	588 533 526	0.57 (0.51; 0.64) 0.56 (0.50; 0.62) 0.56 (0.50; 0.63)	2.90 2.97 2.97	2.50 2.61 2.55

^a^ calculated as ${e}^{- {\beta }_{Xpost} SD\left(Xpre\right)}$ and interpreted as rate ratio of mean net days of sick leave corresponding to a 1 SD decrease in the exposure variable during treatment. Except for work ability that was reversed and calculated as

$${e}^{ {\beta }_{Xpost} SD\left(Xpre\right)}$$.

^b^ Adjusted for the respective exposure variable at pre-treatment

^c^ Further adjusted for pre-treatment levels of gender, age, civil status, yearly income, education level, country of birth, type of work, psychiatric comorbidity, and unemployment

^d^ Further adjusted for pre-treatment levels of all exposure variables

PE: point-estimate; RR: rate ratio; UCL; upper confidence limit

The assumption of linearity between the exposure and the mean of log sick days was assessed by plotting the residuals against the predicted values along with a loess function, showing fairly linear associations between most exposures and log sick days. One exception was the association between perfectionistic concerns and log sick days that showed slight signs of non-linearity, in this case, the estimate should be interpreted as the best linear approximation. Robust standard errors were used to account for the heteroskedasticity of the residuals. The assumption of normally distributed residuals was not evaluated since this assumption has a minor impact on the estimation of coefficients [37]. However, to assess our results’ sensitivity to the error distribution specification, we compared our main results to models using quasi-poisson error distributions. In this sensitivity analysis, the net days of the sick leave variable were rounded to the nearest full day. Estimates were very similar to those from the main models (that used gaussian error distributions). The estimates were within 0.04 on RR scale (using the transformation shown above), except for the association between work ability and sick days, where point estimates were 0.1–0.2 larger, indicating stronger associations compared to the main models. We assessed potential multicollinearity in our models by calculating the variance inflation factor which was < 3 for all exposures (and all other variables), indicating no problems with multicollinearity.

There were no missing data on the outcome variable but some missing data on the exposures and covariates. All analyses were conducted as complete case analyses, the number of available observations for each model is presented in Table 3.

To assess the robustness of the results to unmeasured confounding, we also calculated the E-value for the point estimates, as well as for the confidence limits closest to the null. The E-value measures how much an unmeasured confounder would minimally have to affect both the exposure and the outcome on the risk ratio scale, to reduce the point estimate to the null, or in the case of the confidence limit, shift the confidence interval to include the null [38]. E-values are presented in Table 2.

Analyses were performed using R version 4.1.3.

## Results

We included 880 participants that underwent MMR for exhaustion disorder, with complete data on the number of net days on sick leave in the year following treatment. Most of the participants were 30–53 years (77%), were married or living with a partner (63%) and 86% were female. The pointwise amount of sick leave was reduced for most participants during the follow-up period (Fig. 1). In the year following treatment, the median number of net days of sick leave was 23 days (Q1 = 0; Q3 = 79; max = 366). Mean levels were reduced from pre-treatment to post-treatment for all exposure variables (Table 2).

### Main results

Overall, the main analysis shows that reductions of the exposure levels during treatment were associated with a lower mean of net days of sick leave in the year following treatment (for the WAI estimates were reversed so that lower levels represent better self-perceived work ability) (Table 3). One SD decrease of exhaustion symptoms was associated to 30% (95% CI 34%; 25%) fewer net days of sick leave in the fully adjusted model. The corresponding estimates from the fully adjusted models were: 25% (95% CI: 33%; 16%) fewer net days of sick leave for a one SD decrease in insomnia; 27% (95% CI: 35%; 17%) fewer net days of sick leave for a one SD increase of psychological flexibility; 23% (95% CI: 29%; 14%) fewer net days of sick leave for a one SD decrease of perfectionistic concerns; 23% (95% CI: 31%; 14%) fewer net days of sick leave for a one SD decrease of perfectionistic strivings and; 44% (95% CI: 50%; 37%) fewer net days of sick leave for a one SD increase in perceived work ability.

## Discussion

The present study aimed to examine whether improvements in exhaustion symptoms, insomnia, perfectionism, psychological flexibility, and perceived work ability during treatment of ED were associated with fewer net days of sick leave days in the year following treatment. Overall, large reductions in net sick leave were seen over time in ED patients participating in a standardized CBT-based MMI (Fig. 1). Improvements in all treatment-related variables were associated with fewer net days of sick leave in the year following treatment, with improvements in self-perceived work ability and exhaustion symptoms showing the strongest associations to subsequent sick leave.

The overall large reductions in sick leave documented in this large population of ED patients are encouraging. Our results indicate that participation in a CBT-based MMI with vocational measures is generally followed by reduced sick leave rates. Due to the lack of a control group, we cannot separate the potential effects of MMI from spontaneous recovery or the effects of other factors, such as regulations in the social insurance systems regarding the recommended duration of sick leave due to ED (6–12 months), which could explain the reduced sick leave rates. However, our results also showed that improvements in exhaustion symptoms and other psychological variables targeted in the MMI were associated with lower subsequent sick leave rates. These results are unlikely to be explained by regulations in the social insurance system and were robust to adjustments for other potential confounders. Further, the E-value analyses showed that unmeasured confounders would need to have at least a moderate effect on both the psychological variables and the outcome to explain away these associations. These results strengthen the evidence that improvements in these variables during treatment could reduce sick leave rates in the year following treatment.

Considering previous findings in research on RTW interventions for common mental disorders, it has previously been suggested that while interventions such as CBT may improve symptoms, symptom improvement in and of itself does not necessarily result in reduced sick leave [39, 40]. A reason for this could be that CBT typically focuses on the individual processes assumed to maintain a condition, rather than on the processes of the work context that might influence sick leave outcomes. Even though the current study did include interventions specifically aimed at facilitating RTW, it does show that for ED, improvements in symptoms during CBT-based MMI was associated with fewer net days of sick leave over time. The same can be said of insomnia, as the results from the current study add to previous findings highlighting the importance of acknowledging insomnia in the treatment of ED [41].

Furthermore, while the literature on perfectionism, psychological flexibility, and perceived work ability in relation to ED is limited, the results of the current study show that these variables do indeed appear to be potential targets when aiming to decrease rates of sick leave among patients with ED. Therefore, future clinical research efforts on ED treatment should preferably try to understand how to target perfectionism, psychological flexibility, and perceived work ability more effectively.

It should be noted that while improvements in all psychological variables examined were associated with lower rates of net days of sick leave, the current design does not account for the potential interactions among these variables. A general difficulty in psychology is that constructs tend to overlap, resulting in inadvertent associations between measures. Consequently, little can be said about how improvements in each investigated variable uniquely contributed to decreased sick leave. The same reasoning is transferable to the contents of treatment. The CBT-based multimodal intervention (MMI) employed in the present study consists of multiple components, making it challenging to determine which specific components are associated with improvements. There is currently no consensus regarding psychological theory, methods, or target variables that should be the focus of ED interventions [42]. Therefore, future research could preferably prioritize investigating more focused interventions that are rooted in theoretical models and include specific, quantifiable components with specific targets and outcomes. As reductions in exhaustion symptoms are associated with decreased sick leave rates, refining interventions for ED may also improve RTW outcomes.

### Limitations and strengths

The current study has several strengths. First, this open clinical trial was conducted in a naturalistic setting with permissive inclusion criteria. This strengthens the external validity, and assuming that the results would apply to the broader ED population seems plausible. Second, although a large sample was recruited, drop-out rates were very low, limiting the risk of selection bias. Third, the risk of instrumentation bias was decreased by randomizing the order of the questionnaires at each dispatch. Fourth, we assessed how changes in exhaustion symptoms and psychological variables were associated with later sick leave while controlling for multiple potential confounders. Further, by using E-values, we could also show that the results are somewhat robust to unmeasured confounders. Taken together, we believe that these findings have strengthened the evidence level for a potential causal effect of improvements in exhaustion symptoms and other targeted psychological variables during MMI on subsequent sick leave rates.

The measurement of sick leave data has some limitations. First, sick leave periods shorter than 14 days are not recorded by the Swedish Social Insurance Agency, meaning we do not capture shorter sick leave durations. Second, the measure of net days of sick leave is only a proxy for RTW. While we assume that most of the decreases in sick leave were due to RTW, for some participants, it could also be due to other factors such as voluntary unemployment, studying, discharge from the insurance benefits, or transference to another insurance compensation form. However, as data on disability pension were collected and excluded, we do know that the decrease in net days of sick leave seen in the current paper is not due to early retirement compensation enabled by the social insurance agency. Still, future studies focusing specifically on return to work may benefit from limiting the sample to individuals initially on sick leave and obtaining direct measures on their work attendance. Finally, although we adjusted for several potential confounders, there is still the possibility of residual and unmeasured confounding. For instance, work-related variables, such as work sector, may affect sick leave but were not controlled for in this study.

## Conclusion

In conclusion, this study demonstrates that improvements in exhaustion symptoms, insomnia, perfectionistic behaviors, psychological flexibility, and perceived work ability during CBT-based MMI of ED are associated with lower rates of net days of sick leave in the year following treatment. These findings suggest that interventions that focus on these variables can have a meaningful impact on ED patients’ subsequent sick leave.

### Electronic supplementary material

Below is the link to the electronic supplementary material.

Supplementary Material 1

## Data Availability

The datasets generated and/or analyzed during the current study are not publicly available due to containing patient healthcare data, which may include sensitive information such as health records and sick leave details. However, these datasets can be obtained from the corresponding author upon reasonable request.

## References

[1] American Psychological Association. Stress in America: Stress and Current Events. 2019.

[2] Eurofound. Burnout in the workplace: A review of data and policy responses in the EU [Internet]. Publications Office of the European Union. Luxemburg: Publications Office of theEuropean Union; 2018. 1–41 p. Available from: http://eurofound.link/ef18047.

[3] Hassard J, Teoh KRH, Visockaite G, Dewe P, Cox T. The cost of work-related stress to society: A systematic review. J Occup Health Psychol [Internet]. 2018;23(1):1–17. Available from: http://doi.apa.org/getdoi.cfm?doi=10.1037/ocp0000069. 10.1037/ocp0000069 28358567

[4] Mittendorfer-Rutz E, Kjeldgård L, Runeson B, Perski A, Melchior M, Head J (2012). Sickness absence due to specific Mental Diagnoses and all-cause and cause-specific mortality: a cohort study of 4.9 million Inhabitants of Sweden. PLoS ONE.

[5] Sieurin L, Josephson M, Vingård E. Positive and negative consequences of sick leave for the individual, with special focus on part-time sick leave. Scand J Public Health [Internet]. 2009;37(1):50–6. Available from: http://journals.sagepub.com/doi/10.1177/1403494808097171. 10.1177/1403494808097171 19141555

[6] Eriksson UB, Starrin B, Janson S. Long-Term Sickness Absence Due to Burnout: Absentees’ Experiences. Qual Health Res [Internet]. 2008;18(5):620–32. Available from: http://journals.sagepub.com/doi/10.1177/1049732308316024. 10.1177/1049732308316024 18420536

[7] Grossi G, Perski A, Osika W, Savic I. Stress-related exhaustion disorder – clinical manifestation of burnout? A review of assessment methods, sleep impairments, cognitive disturbances, and neuro‐biological and physiological changes in clinical burnout. Scand J Psychol [Internet]. 2015;56(6):626–36. Available from: https://onlinelibrary.wiley.com/doi/10.1111/sjop.12251. 10.1111/sjop.12251 26496458

[8] Heinemann Lv, Heinemann T. Burnout research: emergence and scientific investigation of a contested diagnosis. Sage Open. 2017;7(1).

[9] Cléach O, Claude, Veil. Vulnérabilités au travail. Naissance et actualité de la psychopathologie du travail. Recueil de textes présentés par Dominique Lhuilier, Toulouse, Erès, 2012. La Nouvelle Revue du Travail [Internet]. 2014;(4). Available from: http://journals.openedition.org/nrt/1765.

[10] Swedish Social Insurance Agency. Sick leave in psychiatric diagnoses. Social insurance report 2020:8. 2020; Available from: www.forsakringskassan.se.

[11] Glise K, Wiegner L, Jonsdottir IH. Long-term follow-up of residual symptoms in patients treated for stress-related exhaustion. BMC Psychol [Internet]. 2020;8(1):26. Available from: https://bmcpsychology.biomedcentral.com/articles/10.1186/s40359-020-0395-8. 10.1186/s40359-020-0395-8 PMC7081527 32188513

[12] Mikkelsen MB, Rosholm M. Systematic review and meta-analysis of interventions aimed at enhancing return to work for sick-listed workers with common mental disorders, stress-related disorders, somatoform disorders and personality disorders. Occupational and Environmental Medicine. Volume 75. BMJ Publishing Group; 2018. pp. 675–86. 10.1136/oemed-2018-105073 29954920

[13] Wallensten J, Åsberg M, Wiklander M, Nager A. Role of rehabilitation in chronic stress-induced exhaustion disorder: A narrative review. J Rehabil Med [Internet]. 2019;51(5):331–42. Available from: https://www.medicaljournals.se/jrm/content/abstract/10.2340/16501977-2545. 10.2340/16501977-2545 30882887

[14] Perski O, Grossi G, Perski A, Niemi M. A systematic review and meta-analysis of tertiary interventions in clinical burnout. Scand J Psychol [Internet]. 2017;58(6):551–61. Available from: http://www.ncbi.nlm.nih.gov/pubmed/29105127. 10.1111/sjop.12398 29105127

[15] Ahola K, Toppinen-Tanner S, Seppänen J. Interventions to alleviate burnout symptoms and to support return to work among employees with burnout: Systematic review and meta-analysis. Burn Res [Internet]. 2017;4:1–11. Available from: https://linkinghub.elsevier.com/retrieve/pii/S2213058616300596.

[16] Finnes A, Ghaderi A, Dahl J, Nager A, Enebrink P. Randomized controlled trial of acceptance and commitment therapy and a workplace intervention for sickness absence due to mental disorders. J Occup Health Psychol [Internet]. 2019;24(1):198–212. Available from: http://doi.apa.org/getdoi.cfm?doi=10.1037/ocp0000097. 10.1037/ocp0000097 28956942

[17] Salomonsson S, Santoft F, Lindsäter E, Ejeby K, Ingvar M, Ljótsson B et al. Effects of cognitive behavioural therapy and return-to‐work intervention for patients on sick leave due to stress‐related disorders: Results from a randomized trial. Scand J Psychol [Internet]. 2020;61(2):281–9. Available from: https://onlinelibrary.wiley.com/doi/10.1111/sjop.12590. 10.1111/sjop.12590 31691305

[18] Hoff A, Fisker J, Poulsen RM, Hjorthøj C, Rosenberg NK, Nordentoft M et al. Integrating vocational rehabilitation and mental healthcare to improve the return-to-work process for people on sick leave with stress-related disorders: results from a randomized trial. Scand J Work Environ Health [Internet]. 2022; Available from: http://www.sjweh.fi/show_abstract.php?abstract_id=4021. 10.5271/sjweh.4021 PMC9527782 35373306

[19] Palmlöf L, Skillgate E, Talbäck M, Josephson M, Vingård E, Holm LW. Poor work ability increases sickness absence over 10 years. Occup Med (Chic Ill) [Internet]. 2019;69(5):359–65. Available from: https://academic.oup.com/occmed/article/69/5/359/5518329. 10.1093/occmed/kqz083 31219583

[20] Selander J, Sun J, Tjulin A, Buys N. Interrelated Factors for Return to Work of Sick-Listed Employees in Sweden. International Journal of Disability Management [Internet]. 2020;15:e7. Available from: https://www.cambridge.org/core/product/identifier/S1833855020000079/type/journal_article.

[21] Stoeber J, Otto K (2006). Positive conceptions of perfectionism: approaches, evidence, Challenges. Personality and Social Psychology Review.

[22] Gulin S, Ellbin S, Jonsdottir IH, Lindqvist Bagge A. Is obsessive–compulsive personality disorder related to stress-related exhaustion? Brain Behav [Internet]. 2021;11(6):1–11. Available from: https://onlinelibrary.wiley.com/doi/10.1002/brb3.2171. 10.1002/brb3.2171 PMC8213937 33969937

[23] Lindsäter E, Axelsson E, Salomonsson S, Santoft F, Ejeby K, Ljótsson B (2018). Internet-based cognitive behavioral therapy for chronic stress: a Randomized Controlled Trial. Psychother Psychosom.

[24] McCracken LM, Morley S. The psychological flexibility model: A basis for integration and progress in psychological approaches to chronic pain management. Journal of Pain [Internet]. 2014;15(3):221–34. 10.1016/j.jpain.2013.10.014. 10.1016/j.jpain.2013.10.014 24581630

[25] Bond FW, Hayes SC, Baer RA, Carpenter KM, Guenole N, Orcutt HK et al. Preliminary Psychometric Properties of the Acceptance and Action Questionnaire–II: A Revised Measure of Psychological Inflexibility and Experiential Avoidance. Behav Ther [Internet]. 2011;42(4):676–88. Available from: https://linkinghub.elsevier.com/retrieve/pii/S0005789411000888. 10.1016/j.beth.2011.03.007 22035996

[26] Melamed S, Kushnir T, Shirom A. Burnout and Risk Factors for Cardiovascular Diseases. Vol. 18, Behavioral medicine (Washington, D.C.). 1992. 53–60 p. 10.1080/08964289.1992.9935172 1392214

[27] van de Leur JC, Buhrman M, Åhs F, Rozental A, Jansen GB. Standardized multimodal intervention for stress-induced exhaustion disorder: an open trial in a clinical setting. BMC Psychiatry [Internet]. 2020;20(1):526. Available from: https://bmcpsychiatry.biomedcentral.com/articles/10.1186/s12888-020-02907-3. 10.1186/s12888-020-02907-3 PMC7643309 33153461

[28] Besèr A, Sorjonen K, Wahlberg K, Peterson U, Nygren Ã, Åsberg M. Construction and evaluation of a self rating scale for stress-induced Exhaustion Disorder, the Karolinska Exhaustion Disorder Scale. Scand J Psychol [Internet]. 2014;55(1):72–82. Available from: https://onlinelibrary.wiley.com/doi/10.1111/sjop.12088. 10.1111/sjop.12088 PMC4235404 24236500

[29] Bastien C, Vallières A, Morin CM. Validation of the Insomnia Severity Index as an outcome measure for insomnia research. Sleep Med [Internet]. 2001;2(4):297–307. Available from: https://linkinghub.elsevier.com/retrieve/pii/S1389945700000654. 10.1016/s1389-9457(00)00065-4 11438246

[30] Lundgren T, Parling T. Swedish Acceptance and Action Questionnaire (SAAQ): a psychometric evaluation. Cogn Behav Ther [Internet]. 2017;46(4):315–26. Available from: https://www.tandfonline.com/doi/full/10.1080/16506073.2016.1250228. 10.1080/16506073.2016.1250228 27931161

[31] van de Parks A, Strååt M, Elfving F, Andersson G, Carlbring P et al. A self-report measure of perfectionism: A confirmatory factor analysis of the Swedish version of the Clinical Perfectionism Questionnaire. Clinical Psychology in Europe [Internet]. 2021;3(4). Available from: https://cpe.psychopen.eu/index.php/cpe/article/view/4581. 10.32872/cpe.4581 PMC9667221 36398287

[32] Ahlstrom L, Grimby-Ekman A, Hagberg M, Dellve L (2010). The work ability index and single-item question: Associations with sick leave, symptoms, and health - A prospective study of women on long-term sick leave. Scand J Work Environ Health.

[33] Virtanen M, Ervasti J, Head J, Oksanen T, Salo P, Pentti J (2018). Lifestyle factors and risk of sickness absence from work: a multicohort study. Lancet Public Health.

[34] Fisker J, Hjorthøj C, Hellström L, Mundy SS, Rosenberg NG, Eplov LF. Predictors of return to work for people on sick leave with common mental disorders: a systematic review and meta-analysis. International Archives of Occupational and Environmental Health. Springer Science and Business Media Deutschland GmbH; 2022. 10.1007/s00420-021-01827-3 35106629

[35] Zigmond AS, Snaith RP. The Hospital Anxiety and Depression Scale. Acta Psychiatr Scand [Internet]. 1983;67(6):361–70. 10.1111/j.1600-0447.1983.tb09716.x. 10.1111/j.1600-0447.1983.tb09716.x 6880820

[36] Nakamura JS, Hong JH, Smith J, Chopik WJ, Chen Y, VanderWeele TJ (2022). Associations between satisfaction with aging and health and well-being outcomes among older US adults. JAMA Netw Open.

[37] Gelman A, Hill J, Vehtari A. Regression and other stories. Cambridge University Press; 2020.

[38] VanderWeele TJ, Ding P (2017). Sensitivity analysis in Observational Research: introducing the E-Value. Ann Intern Med.

[39] Salomonsson S, Santoft F, Lindsäter E, Ejeby K, Ljótsson B, Öst LG et al. Cognitive–behavioural therapy and return-to-work intervention for patients on sick leave due to common mental disorders: a randomised controlled trial. Occup Environ Med [Internet]. 2017;74(12):905–12. Available from: http://oem.bmj.com/content/early/2017/07/28/oemed-2017-104342.abstract. 10.1136/oemed-2017-104342 28756415

[40] Ejeby K, Savitskij R, Ost LG, Ekbom A, Brandt L, Ramnerö J et al. Symptom reduction due to psychosocial interventions is not accompanied by a reduction in sick leave: Results from a randomized controlled trial in primary care. Scand J Prim Health Care [Internet]. 2014;3432(August 2013):1–6. Available from: http://www.ncbi.nlm.nih.gov/pubmed/24742116. 10.3109/02813432.2014.909163 PMC4075019 24742116

[41] Lindsäter E, Axelsson E, Salomonsson S, Santoft F, Ljótsson B, Åkerstedt T et al. The mediating role of insomnia severity in internet-based cognitive behavioral therapy for chronic stress: Secondary analysis of a randomized controlled trial. Behaviour Research and Therapy [Internet]. 2021;136(April 2020):103782. Available from: https://linkinghub.elsevier.com/retrieve/pii/S0005796720302369. 10.1016/j.brat.2020.103782 33276274

[42] van de Leur JC, Jovicic F, Åhslund A, McCracken LM, Buhrman M. Psychological Treatment of Exhaustion Due to Persistent Non-Traumatic Stress: A Scoping Review. Int J Behav Med [Internet]. 2023 Jun 12; Available from: https://link.springer.com/10.1007/s12529-023-10185-y 10.1007/s12529-023-10185-y PMC11001660 37308772

