# Supplementary material for: Associations between improvements in psychological variables and subsequent sick leave among persons receiving a multimodal intervention for exhaustion disorder
Source: BMC Public Health. 2023 Oct 11;23:1976. doi: 10.1186/s12889-023-16799-x (PMC10568869; doi:10.1186/s12889-023-16799-x)
Supplement: Supplementary file 1 — Supplementary Material 1 [file 12889_2023_16799_MOESM1_ESM.docx]

**SUPPLEMENTARY MATERIAL**

**Associations Between Improvements in Psychological Variables and Subsequent Sick Leave Among Persons Receiving a Multimodal Intervention for Exhaustion Disorder**

**Supplemental Table 1.** Pre-treatment characteristics for the full sample, and by quartiles, of exhaustion symptoms (Karolinska Exhaustion Disorder Scale) at post-treatment

**Supplemental Table 2.** Pre-treatment characteristics for the full sample, and by quartiles, of insomnia symptoms (Insomnia Severity Index) at post-treatment

**Supplemental Table 3.** Pre-treatment characteristics for the full sample, and by quartiles, of psychological flexibility (Swedish Acceptance and Action Questionnaire II) at post-treatment

**Supplemental Table 4.** Pre-treatment characteristics for the full sample, and by quartiles, of perfectionistic concerns (subscale of the Clinical Perfectionism Questionnaire) at post-treatment

**Supplemental Table 5.** Pre-treatment characteristics for the full sample, and by quartiles, of perfectionistic strivings (subscale of the Clinical Perfectionism Questionnaire) at post-treatment

**Supplemental Table 6.** Pre-treatment characteristics for the full sample, and by quartiles, of self-perceived work ability (single item from Work Ability Index) at post-treatment

| **Supplemental Table 1.** Pre-treatment characteristics for the full sample, and by quartiles, of exhaustion symptoms (Karolinska Exhaustion Disorder Scale) at post-treatment | | | | | |
| --- | --- | --- | --- | --- | --- |
|  |  | Exhaustions symptoms quartiles at post-treatment | | | |
|  | Full sample  (n = 880) | 1^st^  (n = 230) | 2^nd^  (n = 210) | 3^rd^  (n = 211) | 4^th^  (n = 202) |
| **Gender, n (%)** |  |  |  |  |  |
| Male | 122 (13.9) | 41 (17.8) | 23 (11.0) | 27 (12.8) | 29 (14.4) |
| **Age, n (%)** |  |  |  |  |  |
| 18-29 years | 70 (8.0) | 21 (9.1) | 21 (10.0) | 14 (6.6) | 11 (5.4) |
| 30-41 years | 326 (37.0) | 85 (37.0) | 76 (36.2) | 78 (37.0) | 77 (38.1) |
| 42-53 years | 354 (40.2) | 87 (37.8) | 80 (38.1) | 93 (44.1) | 83 (41.1) |
| 54-65 years | 130 (14.8) | 37 (16.1) | 33 (15.7) | 26 (12.3) | 31 (15.3) |
| **Civil status, n (%)** |  |  |  |  |  |
| Married/living together | 557 (63.3) | 144 (62.6) | 142 (67.6) | 133 (63.0) | 118 (58.4) |
| Partner living apart | 58 (6.6) | 14 (6.1) | 12 (5.7) | 10 (4.7) | 20 (9.9) |
| Single/Other | 265 (30.1) | 72 (31.3) | 56 (26.7) | 68 (32.2) | 64 (31.7) |
| **Yearly household income, n (%)** |  |  |  |  |  |
| 0 – 250k SEK | 73 (8.3) | 16 (7.0) | 12 (5.7) | 18 (8.5) | 26 (12.9) |
| 500k - 1000k SEK | 668 (75.9) | 176 (76.5) | 156 (74.3) | 160 (75.8) | 155 (76.7) |
| >1000k SEK | 139 (15.8) | 38 (16.5) | 42 (20.0) | 33 (15.6) | 21 (10.4) |
| **Education level, n (%)** |  |  |  |  |  |
| Elementary or secondary school | 219 (24.9) | 55 (23.9) | 49 (23.3) | 53 (25.1) | 56 (27.7) |
| University less than 3 years | 140 (15.9) | 39 (17.0) | 36 (17.1) | 26 (12.3) | 34 (16.8) |
| University 3 years or more | 482 (54.8) | 124 (53.9) | 118 (56.2) | 121 (57.3) | 105 (52.0) |
| Other | 39 (4.4) | 12 (5.2) | 7 (3.3) | 11 (5.2) | 7 (3.5) |
| **Country of birth, n (%)** |  |  |  |  |  |
| Sweden | 762 (86.6) | 204 (88.7) | 185 (88.1) | 179 (84.8) | 171 (84.7) |
| The north of Europe | 23 (2.6) | 7 (3.0) | 3 (1.4) | 6 (2.8) | 7 (3.5) |
| Europe | 34 (3.9) | 4 (1.7) | 12 (5.7) | 10 (4.7) | 7 (3.5) |
| Other country | 61 (6.9) | 15 (6.5) | 10 (4.8) | 16 (7.6) | 17 (8.4) |
| **Type of work** |  |  |  |  |  |
| Handling of heavy loads | 19 (2.3) | 6 (2.9) | 3 (1.5) | 3 (1.5) | 7 (3.8) |
| Heavy repetitive work | 40 (4.9) | 6 (2.9) | 10 (5.0) | 11 (5.6) | 13 (7.1) |
| Medium-heavy work | 144 (17.7) | 32 (15.4) | 35 (17.5) | 33 (16.7) | 41 (22.3) |
| Light repetitive work | 49 (6.0) | 13 (6.2) | 11 (5.5) | 17 (8.6) | 7 (3.8) |
| Administrative/computer  work | 562 (69.0) | 151 (72.6) | 141 (70.5) | 134 (67.7) | 116 (63.0) |
| **Comorbidity** |  |  |  |  |  |
| No comorbidity | 528 (60.0) | 144 (62.6) | 122 (58.1) | 140 (66.4) | 104 (51.5) |
| Comorbid psychiatric disorder | 304 (34.5) | 74 (32.2) | 75 (35.7) | 60 (28.4) | 87 (43.1) |
| Comorbid pain disorder | 28 (3.2) | 8 (3.5) | 8 (3.8) | 6 (2.8) | 5 (2.5) |
| Comorbid pain and psychiatric  disorder | 20 (2.3) | 4 (1.7) | 5 (2.4) | 5 (2.4) | 6 (3.0) |
| **Employed** | 786 (93.2) | 208 (92.9) | 191 (95.5) | 185 (93.0) | 178 (91.3) |
| **Net days of sick leave 6 months prior to treatment, mean (SD)** | 91.53 (56.79) | 81.93 (50.56) | 88.10 (55.96) | 96.04 (57.46) | 98.33 (60.80) |
| **Depression symptoms, mean (SD)** | 11.23 (3.68) | 10.20 (3.52) | 11.05 (3.48) | 11.65 (3.75) | 12.23 (3.67) |
| **Anxiety symptoms, mean (SD)** | 11.59 (4.01) | 10.86 (3.89) | 11.22 (3.77) | 11.96 (3.82) | 12.36 (4.36) |

| **Supplemental Table 2.** Pre-treatment characteristics for the full sample, and by quartiles, of insomnia symptoms (Insomnia Severity Index) at post-treatment | | | | | |
| --- | --- | --- | --- | --- | --- |
|  |  | Insomnia symptoms quartiles at post-treatment | | | |
|  | Full sample  (n = 880) | 1^st^  (n = 277) | 2^nd^  (n = 216) | 3^rd^  (n = 218) | 4^th^  (n = 192) |
| **Gender, n (%)** |  |  |  |  |  |
| Male | 122 (13.9) | 35 (15.4) | 25 (11.6) | 29 (13.3) | 30 (15.6) |
| **Age, n (%)** |  |  |  |  |  |
| 18-29 years | 70 (8.0) | 24 (10.6) | 23 (10.6) | 13 (6.0) | 9 (4.7) |
| 30-41 years | 326 (37.0) | 89 (39.2) | 95 (44.0) | 68 (31.2) | 63 (32.8) |
| 42-53 years | 354 (40.2) | 85 (37.4) | 72 (33.3) | 96 (44.0) | 89 (46.4) |
| 54-65 years | 130 (14.8) | 29 (12.8) | 26 (12.0) | 41 (18.8) | 31 (16.1) |
| **Civil status, n (%)** |  |  |  |  |  |
| Married/living together | 557 (63.3) | 156 (68.7) | 134 (62.0) | 146 (67.0) | 101 (52.6) |
| Partner living apart | 58 (6.6) | 11 (4.8) | 12 (5.6) | 15 (6.9) | 18 (9.4) |
| Single/Other | 265 (30.1) | 60 (26.4) | 70 (32.4) | 57 (26.1) | 73 (38.0) |
| **Yearly household income, n (%)** |  |  |  |  |  |
| 0 – 250k SEK | 73 (8.3) | 18 (7.9) | 15 (6.9) | 15 (6.9) | 24 (12.5) |
| 500k - 1000k SEK | 668 (75.9) | 168 (74.0) | 167 (77.3) | 169 (77.5) | 144 (75.0) |
| >1000k SEK | 139 (15.8) | 41 (18.1) | 34 (15.7) | 34 (15.6) | 24 (12.5) |
| **Education level, n (%)** |  |  |  |  |  |
| Elementary or secondary school | 219 (24.9) | 64 (28.2) | 47 (21.8) | 54 (24.8) | 47 (24.5) |
| University less than 3 years | 140 (15.9) | 32 (14.1) | 31 (14.4) | 38 (17.4) | 35 (18.2) |
| University 3 years or more | 482 (54.8) | 123 (54.2) | 126 (58.3) | 116 (53.2) | 103 (53.6) |
| Other | 39 (4.4) | 8 (3.5) | 12 (5.6) | 10 (4.6) | 7 (3.6) |
| **Country of birth, n (%)** |  |  |  |  |  |
| Sweden | 762 (86.6) | 201 (88.5) | 190 (88.0) | 193 (88.5) | 154 (80.2) |
| The north of Europe | 23 (2.6) | 6 (2.6) | 7 (3.2) | 6 (2.8) | 4 (2.1) |
| Europe | 34 (3.9) | 6 (2.6) | 6 (2.8) | 9 (4.1) | 12 (6.2) |
| Other country | 61 (6.9) | 14 (6.2) | 13 (6.0) | 10 (4.6) | 22 (11.5) |
| **Type of work** |  |  |  |  |  |
| Handling of heavy loads | 19 (2.3) | 4 (1.9) | 5 (2.5) | 4 (2.0) | 6 (3.4) |
| Heavy repetitive work | 40 (4.9) | 13 (6.2) | 8 (4.0) | 8 (4.0) | 11 (6.1) |
| Medium-heavy work | 144 (17.7) | 31 (14.8) | 42 (20.8) | 31 (15.7) | 37 (20.7) |
| Light repetitive work | 49 (6.0) | 18 (8.6) | 13 (6.4) | 9 (4.5) | 8 (4.5) |
| Administrative/computer  work | 562 (69.0) | 144 (68.6) | 134 (66.3) | 146 (73.7) | 117 (65.4) |
| **Comorbidity** |  |  |  |  |  |
| No comorbidity | 528 (60.0) | 145 (63.9) | 134 (62.0) | 132 (60.6) | 98 (51.0) |
| Comorbid psychiatric disorder | 304 (34.5) | 74 (32.6) | 69 (31.9) | 74 (33.9) | 80 (41.7) |
| Comorbid pain disorder | 28 (3.2) | 3 (1.3) | 11 (5.1) | 6 (2.8) | 7 (3.6) |
| Comorbid pain and psychiatric  disorder | 20 (2.3) | 5 (2.2) | 2 (0.9) | 6 (2.8) | 7 (3.6) |
| **Employed** | 786 (93.2) | 204 (92.7) | 200 (93.9) | 188 (92.6) | 170 (93.4) |
| **Net days of sick leave 6 months prior to treatment, mean (SD)** | 91.53 (56.79) | 89.89 (54.57) | 88.18 (55.32) | 93.42 (57.46) | 92.33 (59.41) |
| **Depression symptoms, mean (SD)** | 11.23 (3.68) | 10.81 (3.75) | 10.97 (3.54) | 11.51 (3.64) | 11.79 (3.71) |
| **Anxiety symptoms, mean (SD)** | 11.59 (4.01) | 11.03 (4.01) | 11.20 (3.83) | 11.44 (4.02) | 12.81 (3.97) |

| **Supplemental Table 3.** Pre-treatment characteristics for the full sample, and by quartiles, of psychological flexibility (Swedish Acceptance and Action Questionnaire II) at post-treatment | | | | | |
| --- | --- | --- | --- | --- | --- |
|  |  | Psychological flexibility quartiles at post-treatment | | | |
|  | Full sample  (n = 880) | 1^st^  (n = 215) | 2^nd^  (n = 252) | 3^rd^  (n = 203) | 4^th^  (n = 182) |
| **Gender, n (%)** |  |  |  |  |  |
| Male | 122 (13.9) | 28 (13.0) | 32 (12.7) | 25 (12.3) | 34 (18.7) |
| **Age, n (%)** |  |  |  |  |  |
| 18-29 years | 70 (8.0) | 13 (6.0) | 25 (9.9) | 17 (8.4) | 14 (7.7) |
| 30-41 years | 326 (37.0) | 72 (33.5) | 93 (36.9) | 87 (42.9) | 64 (35.2) |
| 42-53 years | 354 (40.2) | 90 (41.9) | 102 (40.5) | 72 (35.5) | 77 (42.3) |
| 54-65 years | 130 (14.8) | 40 (18.6) | 32 (12.7) | 27 (13.3) | 27 (14.8) |
| **Civil status, n (%)** |  |  |  |  |  |
| Married/living together | 557 (63.3) | 144 (67.0) | 171 (67.9) | 129 (63.5) | 93 (51.1) |
| Partner living apart | 58 (6.6) | 6 (2.8) | 17 (6.7) | 15 (7.4) | 18 (9.9) |
| Single/Other | 265 (30.1) | 65 (30.2) | 64 (25.4) | 59 (29.1) | 71 (39.0) |
| **Yearly household income, n (%)** |  |  |  |  |  |
| 0 – 250k SEK | 73 (8.3) | 14 (6.5) | 18 (7.1) | 13 (6.4) | 27 (14.8) |
| 500k - 1000k SEK | 668 (75.9) | 164 (76.3) | 182 (72.2) | 168 (82.8) | 132 (72.5) |
| >1000k SEK | 139 (15.8) | 37 (17.2) | 52 (20.6) | 22 (10.8) | 23 (12.6) |
| **Education level, n (%)** |  |  |  |  |  |
| Elementary or secondary school | 219 (24.9) | 60 (27.9) | 61 (24.2) | 43 (21.2) | 47 (25.8) |
| University less than 3 years | 140 (15.9) | 30 (14.0) | 40 (15.9) | 34 (16.7) | 31 (17.0) |
| University 3 years or more | 482 (54.8) | 114 (53.0) | 140 (55.6) | 117 (57.6) | 98 (53.8) |
| Other | 39 (4.4) | 11 (5.1) | 11 (4.4) | 9 (4.4) | 6 (3.3) |
| **Country of birth, n (%)** |  |  |  |  |  |
| Sweden | 762 (86.6) | 188 (87.4) | 221 (87.7) | 173 (85.2) | 156 (85.7) |
| The north of Europe | 23 (2.6) | 5 (2.3) | 8 (3.2) | 5 (2.5) | 4 (2.2) |
| Europe | 34 (3.9) | 5 (2.3) | 10 (4.0) | 9 (4.4) | 9 (4.9) |
| Other country | 61 (6.9) | 17 (7.9) | 13 (5.2) | 16 (7.9) | 13 (7.1) |
| **Type of work** |  |  |  |  |  |
| Handling of heavy loads | 19 (2.3) | 3 (1.5) | 4 (1.7) | 8 (4.2) | 4 (2.5) |
| Heavy repetitive work | 40 (4.9) | 8 (4.0) | 14 (6.0) | 6 (3.1) | 12 (7.4) |
| Medium-heavy work | 144 (17.7) | 45 (22.5) | 41 (17.5) | 28 (14.7) | 26 (16.0) |
| Light repetitive work | 49 (6.0) | 7 (3.5) | 19 (8.1) | 10 (5.2) | 11 (6.7) |
| Administrative/computer  work | 562 (69.0) | 137 (68.5) | 156 (66.7) | 139 (72.8) | 110 (67.5) |
| **Comorbidity** |  |  |  |  |  |
| No comorbidity | 528 (60.0) | 152 (70.7) | 143 (56.7) | 127 (62.6) | 89 (48.9) |
| Comorbid psychiatric disorder | 304 (34.5) | 53 (24.7) | 94 (37.3) | 63 (31.0) | 85 (46.7) |
| Comorbid pain disorder | 28 (3.2) | 8 (3.7) | 12 (4.8) | 6 (3.0) | 1 (0.5) |
| Comorbid pain and psychiatric  disorder | 20 (2.3) | 2 (0.9) | 3 (1.2) | 7 (3.4) | 7 (3.8) |
| **Employed** | 786 (93.2) | 192 (94.1) | 228 (95.4) | 184 (94.4) | 157 (87.7) |
| **Net days of sick leave 6 months prior to treatment, mean (SD)** | 91.53 (56.79) | 91.61 (55.38) | 89.24 (56.45) | 92.44 (55.16) | 91.17 (59.01) |
| **Depression symptoms, mean (SD)** | 11.23 (3.68) | 9.91 (3.70) | 11.25 (3.41) | 11.52 (3.44) | 12.57 (3.75) |
| **Anxiety symptoms, mean (SD)** | 11.59 (4.01) | 9.70 (4.13) | 11.57 (3.69) | 12.34 (3.61) | 13.00 (3.82) |

| **Supplemental Table 4.** Pre-treatment characteristics for the full sample, and by quartiles, of perfectionistic concerns (subscale of the Clinical Perfectionism Questionnaire) at post-treatment | | | | | |
| --- | --- | --- | --- | --- | --- |
|  |  | Perfectionistic concerns quartiles at post-treatment | | | |
|  | Full sample  (n = 880) | 1^st^  (n = 235) | 2^nd^  (n = 261) | 3^rd^  (n = 156) | 4^th^  (n = 203) |
| **Gender, n (%)** |  |  |  |  |  |
| Male | 122 (13.9) | 30 (12.8) | 35 (13.4) | 28 (17.9) | 27 (13.3) |
| **Age, n (%)** |  |  |  |  |  |
| 18-29 years | 70 (8.0) | 16 (6.8) | 19 (7.3) | 15 (9.6) | 19 (9.4) |
| 30-41 years | 326 (37.0) | 76 (32.3) | 97 (37.2) | 59 (37.8) | 83 (40.9) |
| 42-53 years | 354 (40.2) | 93 (39.6) | 112 (42.9) | 63 (40.4) | 76 (37.4) |
| 54-65 years | 130 (14.8) | 50 (21.3) | 33 (12.6) | 19 (12.2) | 25 (12.3) |
| **Civil status, n (%)** |  |  |  |  |  |
| Married/living together | 557 (63.3) | 139 (59.1) | 169 (64.8) | 107 (68.6) | 123 (60.6) |
| Partner living apart | 58 (6.6) | 16 (6.8) | 17 (6.5) | 6 (3.8) | 17 (8.4) |
| Single/Other | 265 (30.1) | 80 (34.0) | 75 (28.7) | 43 (27.6) | 63 (31.0) |
| **Yearly household income, n (%)** |  |  |  |  |  |
| 0 – 250k SEK | 73 (8.3) | 21 (8.9) | 16 (6.1) | 13 (8.3) | 22 (10.8) |
| 500k - 1000k SEK | 668 (75.9) | 174 (74.0) | 205 (78.5) | 119 (76.3) | 151 (74.4) |
| >1000k SEK | 139 (15.8) | 40 (17.0) | 40 (15.3) | 24 (15.4) | 30 (14.8) |
| **Education level, n (%)** |  |  |  |  |  |
| Elementary or secondary school | 219 (24.9) | 58 (24.7) | 64 (24.5) | 45 (28.8) | 46 (22.7) |
| University less than 3 years | 140 (15.9) | 38 (16.2) | 48 (18.4) | 18 (11.5) | 32 (15.8) |
| University 3 years or more | 482 (54.8) | 124 (52.8) | 143 (54.8) | 86 (55.1) | 116 (57.1) |
| Other | 39 (4.4) | 15 (6.4) | 6 (2.3) | 7 (4.5) | 9 (4.4) |
| **Country of birth, n (%)** |  |  |  |  |  |
| Sweden | 762 (86.6) | 205 (87.2) | 221 (84.7) | 138 (88.5) | 176 (86.7) |
| The north of Europe | 23 (2.6) | 6 (2.6) | 7 (2.7) | 4 (2.6) | 6 (3.0) |
| Europe | 34 (3.9) | 7 (3.0) | 12 (4.6) | 6 (3.8) | 8 (3.9) |
| Other country | 61 (6.9) | 17 (7.2) | 21 (8.0) | 8 (5.1) | 13 (6.4) |
| **Type of work** |  |  |  |  |  |
| Handling of heavy loads | 19 (2.3) | 6 (2.8) | 2 (0.8) | 5 (3.5) | 6 (3.2) |
| Heavy repetitive work | 40 (4.9) | 9 (4.2) | 16 (6.5) | 9 (6.4) | 6 (3.2) |
| Medium-heavy work | 144 (17.7) | 38 (17.6) | 48 (19.4) | 20 (14.2) | 34 (18.2) |
| Light repetitive work | 49 (6.0) | 10 (4.6) | 17 (6.9) | 12 (8.5) | 9 (4.8) |
| Administrative/computer  work | 562 (69.0) | 153 (70.8) | 164 (66.4) | 95 (67.4) | 132 (70.6) |
| **Comorbidity** |  |  |  |  |  |
| No comorbidity | 528 (60.0) | 156 (66.4) | 155 (59.4) | 97 (62.2) | 103 (50.7) |
| Comorbid psychiatric disorder | 304 (34.5) | 68 (28.9) | 85 (32.6) | 55 (35.3) | 89 (43.8) |
| Comorbid pain disorder | 28 (3.2) | 7 (3.0) | 12 (4.6) | 2 (1.3) | 6 (3.0) |
| Comorbid pain and psychiatric  disorder | 20 (2.3) | 4 (1.7) | 9 (3.4) | 2 (1.3) | 5 (2.5) |
| **Employed** | 786 (93.2) | 212 (94.2) | 243 (96.8) | 136 (90.1) | 173 (89.6) |
| **Net days of sick leave 6 months prior to treatment, mean (SD)** | 91.53 (56.79) | 92.77 (53.78) | 91.86 (55.34) | 91.97 (62.04) | 86.68 (56.42) |
| **Depression symptoms, mean (SD)** | 11.23 (3.68) | 10.38 (3.94) | 11.39 (3.42) | 11.33 (3.50) | 12.01 (3.66) |
| **Anxiety symptoms, mean (SD)** | 11.59 (4.01) | 10.12 (4.29) | 11.73 (3.67) | 12.09 (3.74) | 12.66 (3.83) |

| **Supplemental Table 5.** Pre-treatment characteristics for the full sample, and by quartiles, of perfectionistic strivings (subscale of the Clinical Perfectionism Questionnaire) at post-treatment | | | | | |
| --- | --- | --- | --- | --- | --- |
|  |  | Perfectionistic strivings quartiles at post-treatment | | | |
|  | Full sample  (n = 880) | 1^st^  (n = 235) | 2^nd^  (n = 261) | 3^rd^  (n = 156) | 4^th^  (n = 203) |
| **Gender, n (%)** |  |  |  |  |  |
| Male | 122 (13.9) | 30 (12.8) | 35 (13.4) | 28 (17.9) | 27 (13.3) |
| **Age, n (%)** |  |  |  |  |  |
| 18-29 years | 70 (8.0) | 16 (6.8) | 19 (7.3) | 15 (9.6) | 19 (9.4) |
| 30-41 years | 326 (37.0) | 76 (32.3) | 97 (37.2) | 59 (37.8) | 83 (40.9) |
| 42-53 years | 354 (40.2) | 93 (39.6) | 112 (42.9) | 63 (40.4) | 76 (37.4) |
| 54-65 years | 130 (14.8) | 50 (21.3) | 33 (12.6) | 19 (12.2) | 25 (12.3) |
| **Civil status, n (%)** |  |  |  |  |  |
| Married/living together | 557 (63.3) | 139 (59.1) | 169 (64.8) | 107 (68.6) | 123 (60.6) |
| Partner living apart | 58 (6.6) | 16 (6.8) | 17 (6.5) | 6 (3.8) | 17 (8.4) |
| Single/Other | 265 (30.1) | 80 (34.0) | 75 (28.7) | 43 (27.6) | 63 (31.0) |
| **Yearly household income, n (%)** |  |  |  |  |  |
| 0 – 250k SEK | 73 (8.3) | 21 (8.9) | 16 (6.1) | 13 (8.3) | 22 (10.8) |
| 500k - 1000k SEK | 668 (75.9) | 174 (74.0) | 205 (78.5) | 119 (76.3) | 151 (74.4) |
| >1000k SEK | 139 (15.8) | 40 (17.0) | 40 (15.3) | 24 (15.4) | 30 (14.8) |
| **Education level, n (%)** |  |  |  |  |  |
| Elementary or secondary school | 219 (24.9) | 58 (24.7) | 64 (24.5) | 45 (28.8) | 46 (22.7) |
| University less than 3 years | 140 (15.9) | 38 (16.2) | 48 (18.4) | 18 (11.5) | 32 (15.8) |
| University 3 years or more | 482 (54.8) | 124 (52.8) | 143 (54.8) | 86 (55.1) | 116 (57.1) |
| Other | 39 (4.4) | 15 (6.4) | 6 (2.3) | 7 (4.5) | 9 (4.4) |
| **Country of birth, n (%)** |  |  |  |  |  |
| Sweden | 762 (86.6) | 205 (87.2) | 221 (84.7) | 138 (88.5) | 176 (86.7) |
| The north of Europe | 23 (2.6) | 6 (2.6) | 7 (2.7) | 4 (2.6) | 6 (3.0) |
| Europe | 34 (3.9) | 7 (3.0) | 12 (4.6) | 6 (3.8) | 8 (3.9) |
| Other country | 61 (6.9) | 17 (7.2) | 21 (8.0) | 8 (5.1) | 13 (6.4) |
| **Type of work** |  |  |  |  |  |
| Handling of heavy loads | 19 (2.3) | 6 (2.8) | 2 (0.8) | 5 (3.5) | 6 (3.2) |
| Heavy repetitive work | 40 (4.9) | 9 (4.2) | 16 (6.5) | 9 (6.4) | 6 (3.2) |
| Medium-heavy work | 144 (17.7) | 38 (17.6) | 48 (19.4) | 20 (14.2) | 34 (18.2) |
| Light repetitive work | 49 (6.0) | 10 (4.6) | 17 (6.9) | 12 (8.5) | 9 (4.8) |
| Administrative/computer  work | 562 (69.0) | 153 (70.8) | 164 (66.4) | 95 (67.4) | 132 (70.6) |
| **Comorbidity** |  |  |  |  |  |
| No comorbidity | 528 (60.0) | 156 (66.4) | 155 (59.4) | 97 (62.2) | 103 (50.7) |
| Comorbid psychiatric disorder | 304 (34.5) | 68 (28.9) | 85 (32.6) | 55 (35.3) | 89 (43.8) |
| Comorbid pain disorder | 28 (3.2) | 7 (3.0) | 12 (4.6) | 2 (1.3) | 6 (3.0) |
| Comorbid pain and psychiatric  disorder | 20 (2.3) | 4 (1.7) | 9 (3.4) | 2 (1.3) | 5 (2.5) |
| **Employed** | 786 (93.2) | 212 (94.2) | 243 (96.8) | 136 (90.1) | 173 (89.6) |
| **Net days of sick leave 6 months prior to treatment, mean (SD)** | 91.53 (56.79) | 92.77 (53.78) | 91.86 (55.34) | 91.97 (62.04) | 86.68 (56.42) |
| **Depression symptoms, mean (SD)** | 11.23 (3.68) | 10.38 (3.94) | 11.39 (3.42) | 11.33 (3.50) | 12.01 (3.66) |
| **Anxiety symptoms, mean (SD)** | 11.59 (4.01) | 10.12 (4.29) | 11.73 (3.67) | 12.09 (3.74) | 12.66 (3.83) |

| **Supplemental Table 6.** Pre-treatment characteristics for the full sample, and by quartiles, of self-perceived work ability (single item from Work Ability Index) at post-treatment | | | | | |
| --- | --- | --- | --- | --- | --- |
|  |  | Self-perceived work ability quartiles at post-treatment | | | |
|  | Full sample  (n = 880) | 1^st^  (n = 191) | 2^nd^  (n = 202) | 3^rd^  (n = 120) | 4^th^  (n = 77) |
| **Gender, n (%)** |  |  |  |  |  |
| Male | 122 (13.9) | 22 (11.5) | 37 (18.3) | 16 (13.3) | 9 (11.7) |
| **Age, n (%)** |  |  |  |  |  |
| 18-29 years | 70 (8.0) | 11 (5.8) | 18 (8.9) | 14 (11.7) | 4 (5.2) |
| 30-41 years | 326 (37.0) | 72 (37.7) | 75 (37.1) | 47 (39.2) | 26 (33.8) |
| 42-53 years | 354 (40.2) | 78 (40.8) | 85 (42.1) | 41 (34.2) | 35 (45.5) |
| 54-65 years | 130 (14.8) | 30 (15.7) | 24 (11.9) | 18 (15.0) | 12 (15.6) |
| **Civil status, n (%)** |  |  |  |  |  |
| Married/living together | 557 (63.3) | 117 (61.3) | 132 (65.3) | 72 (60.0) | 45 (58.4) |
| Partner living apart | 58 (6.6) | 16 (8.4) | 16 (7.9) | 5 (4.2) | 9 (11.7) |
| Single/Other | 265 (30.1) | 58 (30.4) | 54 (26.7) | 43 (35.8) | 23 (29.9) |
| **Yearly household income, n (%)** |  |  |  |  |  |
| 0 – 250k SEK | 73 (8.3) | 21 (11.0) | 12 (5.9) | 11 (9.2) | 2 (2.6) |
| 500k - 1000k SEK | 668 (75.9) | 151 (79.1) | 151 (74.8) | 94 (78.3) | 59 (76.6) |
| >1000k SEK | 139 (15.8) | 19 (9.9) | 39 (19.3) | 15 (12.5) | 16 (20.8) |
| **Education level, n (%)** |  |  |  |  |  |
| Elementary or secondary school | 219 (24.9) | 45 (23.6) | 47 (23.3) | 29 (24.2) | 14 (18.2) |
| University less than 3 years | 140 (15.9) | 28 (14.7) | 29 (14.4) | 24 (20.0) | 12 (15.6) |
| University 3 years or more | 482 (54.8) | 113 (59.2) | 117 (57.9) | 62 (51.7) | 48 (62.3) |
| Other | 39 (4.4) | 5 (2.6) | 9 (4.5) | 5 (4.2) | 3 (3.9) |
| **Country of birth, n (%)** |  |  |  |  |  |
| Sweden | 762 (86.6) | 168 (88.0) | 179 (88.6) | 99 (82.5) | 68 (88.3) |
| The north of Europe | 23 (2.6) | 6 (3.1) | 5 (2.5) | 2 (1.7) | 2 (2.6) |
| Europe | 34 (3.9) | 5 (2.6) | 8 (4.0) | 10 (8.3) | 2 (2.6) |
| Other country | 61 (6.9) | 12 (6.3) | 10 (5.0) | 9 (7.5) | 5 (6.5) |
| **Type of work** |  |  |  |  |  |
| Handling of heavy loads | 19 (2.3) | 4 (2.3) | 4 (2.1) | 3 (2.6) | 2 (2.7) |
| Heavy repetitive work | 40 (4.9) | 13 (7.6) | 4 (2.1) | 7 (6.1) | 3 (4.1) |
| Medium-heavy work | 144 (17.7) | 29 (17.0) | 28 (14.9) | 19 (16.5) | 13 (17.8) |
| Light repetitive work | 49 (6.0) | 13 (7.6) | 14 (7.4) | 6 (5.2) | 2 (2.7) |
| Administrative/computer  work | 562 (69.0) | 112 (65.5) | 138 (73.4) | 80 (69.6) | 53 (72.6) |
| **Comorbidity** |  |  |  |  |  |
| No comorbidity | 528 (60.0) | 111 (58.1) | 122 (60.4) | 85 (70.8) | 46 (59.7) |
| Comorbid psychiatric disorder | 304 (34.5) | 72 (37.7) | 70 (34.7) | 33 (27.5) | 26 (33.8) |
| Comorbid pain disorder | 28 (3.2) | 2 (1.0) | 7 (3.5) | 2 (1.7) | 4 (5.2) |
| Comorbid pain and psychiatric  disorder | 20 (2.3) | 6 (3.1) | 3 (1.5) | 0 (0.0) | 1 (1.3) |
| **Employed** | 786 (93.2) | 169 (90.9) | 184 (92.9) | 112 (95.7) | 74 (96.1) |
| **Net days of sick leave 6 months prior to treatment, mean (SD)** | 91.53 (56.79) | 108.23 (58.38) | 85.98 (56.51) | 74.07 (52.84) | 82.46 (48.10) |
| **Depression symptoms, mean (SD)** | 11.23 (3.68) | 11.60 (3.76) | 11.62 (3.69) | 10.43 (3.24) | 10.79 (3.71) |
| **Anxiety symptoms, mean (SD)** | 11.59 (4.01) | 11.86 (4.44) | 11.71 (3.86) | 11.53 (3.88) | 11.31 (3.72) |
